# Clinicians’ Guide to Artificial Intelligence in Colon Capsule Endoscopy—Technology Made Simple

**DOI:** 10.3390/diagnostics13061038

**Published:** 2023-03-08

**Authors:** Ian I. Lei, Gohar J. Nia, Elizabeth White, Hagen Wenzek, Santi Segui, Angus J. M. Watson, Anastasios Koulaouzidis, Ramesh P. Arasaradnam

**Affiliations:** 1 Department of Gastroenterology, University Hospital of Coventry and Warwickshire, Coventry CV2 2DX, UK; brian.lei@uhcw.nhs.uk (I.I.L.);; 2 CorporateHealth International, Inverness IV2 5NA, UK; 3 Mathematics and Computer Science Department, The University of Barcelona, 58508007 Barcelona, Spain; 4 Institute of Applied Health Sciences, University of Aberdeen, Aberdeen AB24 3FX, UK; 5 Department of Gastroenterology, Odense University Hospital & Svendborg Sygehus, 5700 Odense, Denmark; 6 Department of Clinical Research, University of Southern Denmark (SDU), 5000 Odense, Denmark; 7 Department of Social Medicine and Public Health, Pomeranian Medical University, 70-204 Szczecin, Poland; 8 Warwick Medical School, University of Warwick, Coventry CV4 7AL, UK; 9 Department of Gastroenterology, Leicester Cancer Centre, University of Leicester, Leicester LE1 7RH, UK

**Keywords:** artificial intelligence (AI), machine learning (ML), deep learning (DL), convolutional neural networks (CNN), decision-making systems (DMS), colon capsule endoscopy (CCE)

## Abstract

Artificial intelligence (AI) applications have become widely popular across the healthcare ecosystem. Colon capsule endoscopy (CCE) was adopted in the NHS England pilot project following the recent COVID pandemic’s impact. It demonstrated its capability to relieve the national backlog in endoscopy. As a result, AI-assisted colon capsule video analysis has become gastroenterology’s most active research area. However, with rapid AI advances, mastering these complex machine learning concepts remains challenging for healthcare professionals. This forms a barrier for clinicians to take on this new technology and embrace the new era of big data. This paper aims to bridge the knowledge gap between the current CCE system and the future, fully integrated AI system. The primary focus is on simplifying the technical terms and concepts in machine learning. This will hopefully address the general “fear of the unknown in AI” by helping healthcare professionals understand the basic principle of machine learning in capsule endoscopy and apply this knowledge in their future interactions and adaptation to AI technology. It also summarises the evidence of AI in CCE and its impact on diagnostic pathways. Finally, it discusses the unintended consequences of using AI, ethical challenges, potential flaws, and bias within clinical settings.

## 1. Introduction

The recent decade’s profound technological advances have considerably transformed the medical landscape. Artificial intelligence (AI) applications have become widely popular in genomic analysis, robotic surgery, prediction and support diagnosis, and treatment decision-making across the healthcare ecosystem. There has also been significant interest in AI solutions in gastroenterology in recent years. With many studies published and potential opportunities available in this field, gastroenterology and endoscopy healthcare professionals must understand and evaluate AI studies as critical stakeholders in successfully developing and implementing AI technologies. 

Colon capsule endoscopy (CCE) was first tested in 2006 with the first multicentre study published in Israel [1]. Compared with the gold (reference) standard, i.e., colonoscopy, CCE was first met with scepticism due to its disadvantages, including extensive bowel preparation to achieve a reasonable polyp detection rate (PDR), high cost, and inability to perform biopsy or therapy (e.g., polypectomy). Even though the PillCam^®^Colon2 was upgraded to allow panoramic views in 2011, the uptake of CCE could have been better in the UK. However, following the impact of the recent COVID pandemic, an NHS Scotland evaluation demonstrated that the technology could lead to relieving the backlog in endoscopy on a national level. Still, CCE generates a video containing more than 50,000 images; this could be time-consuming and inefficient to analyse [2,3]. As a result, the advances in AI application on image analysis make AI-assisted CCE video analysis one of the most active research areas.

Nowadays, it is broadly accepted that the data generation rate is beyond the human cognitive capacity to be effectively analysed and managed. Therefore, AI will likely have a complementary role in delivering healthcare services soon. Nonetheless, due to the complexity of machine learning (ML), mastering the concept of AI by clinicians remains challenging [4,5,6,7].

Robust research into computer-aided detection (CAD) in upper and lower gastrointestinal (GI) endoscopy images has demonstrated encouraging results in recent years [8,9]. The success also became visible in the wireless capsule endoscopy (WCE) field, where an early CAD model on WCE showed a sensitivity of 88.2%, specificity of 90.9%, and accuracy of 90.8% to detect erosions and ulcerations, with evidence of relieving the reader’s overall workload and reading time [10,11]. This revived interest is also being transferred into the CCE world. AI started to be used for various tasks and achieved the first remarkable results: a recent meta-analysis showed that the sensitivities were 86.5–95.5% for bowel-cleansing assessment and 47.4–98.1% for the detection of colorectal cancer (CRC) and polyps [12,13].

Understandably, the predominant focus in the literature is on the evidence around the accuracy of these AI models in CCE, as the authors’ goal was undoubtedly to build trust around artificial intelligence in the clinical world. However, to encourage the adoption of CCE AI technology in a clinical setting, understanding the “how” in addition to any data-driven evidence is essential to build that trust among clinical professionals. Therefore, this paper uses a different approach to bridge the gap between recognition and trust. We first simplify technical terms and then focus on how existing evidence of AI in CCE shows its impact on diagnostic pathways. We also highlight the unintended consequences of using AI, potential flaws, and bias within clinical settings.

The ultimate aim is a seamless collaboration of medical professionals and computer scientists to translate prototype AI solutions more quickly into valuable clinical tools.

## 2. AI Terminology and the Concept of Machine Learning

### 2.1. The Difference between Machine Learning and Artificial Intelligence 

The public has used AI interchangeably with machine learning (ML), which refers to using computers to model intelligent behaviour that can perform tasks. However, AI is commonly defined as the development of machine capabilities to simulate natural intelligence by performing tasks that usually require human input.

On the other hand, ML is a subfield of AI that uses a combination of mathematical and statistical modelling techniques that utilise a variety of algorithms to learn and automatically improve the prediction of the result. It aims to build mathematical models based on the given data that have predictive power over new and unseen data [14]. The difference is that AI relates only to creating intelligent models that can simulate human cognition, whereas ML is the subset of AI that allows models to learn from data.

### 2.2. Terminology in Machine Learning

To understand and apply the complex technical science of ML in CCE, it is essential to start by addressing the terminology gap in computing engineering for medical professionals. This could ensure that all the concepts are understood correctly. Furthermore, sharing jargon and expertise from both fields can narrow this communication gap. Therefore, we provide the most basic and relevant technical terminology in machine learning for all medical professionals (Table 1).

### 2.3. Machine Learning

**Figure 1:**
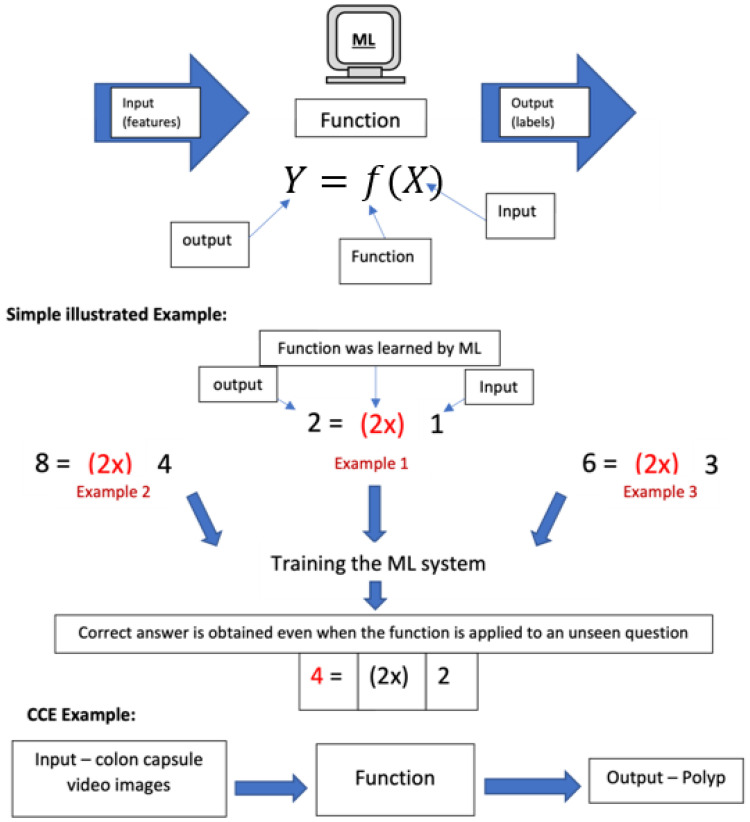
This shows the process of transforming the input into the output by using a function that programmers in computer science create. On the other hand, the machine learning system can learn and develop the function by studying the existing input–output pairs to build a perfect function without relying on the programmer. Examples are included to demonstrate the basic concept of machine learning by using a simple mathematical function.

ML is similar to computer programming, as illustrated in Figure 1. The process of transforming the input into the output is known as a function. In computer programming, the programmer encodes the steps based on rules into functions to provide an automated output. Manual efforts are required to support this rule-based technique.

In contrast, that function correlating the input and output remains unknown in ML. Instead of relying on a programmer to find the function, the ML system can learn and create the function by studying the existing input–output pairs via training. After training the machine learning system on numerous input–output pairs, it will build a function that can accurately process the unseen input data (features) to an accurately predicted output data (prediction) (Figure 1). Therefore, one of the advantages of using machine learning is that it can learn and develop a tremendously complex function based on the multitude of input–output pairs, which would be impractical or impossible for a programmer to achieve [15].

An equivalent analogy would be the process of learning how to drive a manual car. First, the learner is taught the basic principles, including highway codes, gear shifting, driving, parking etc. (examples). For example, when starting a car from a still to a moving position, the driver must shift the gear and apply pressure on the gas paddle (this is the essential function learned from examples). Then, the learner, taught by the expert instructor, repeatedly trains in various preplanned weather, roads, and roundabout (learning and improving the function) during their training (training loops). Once these basic skills and principles are discovered, the new driver can drive different types of cars on any previously unseen roads and areas (new unrecognised input) with further assistance from the driving instructor (validation sets). The driving skills will continue to improve until they are adequate for the driving test (e.g., the test dataset). When more types of different roads, roundabouts, and countryside roads are explored through the driving process, the driving skills are improved continuously (exposed to an extensive dataset to improve the overall function in retraining after the test dataset).

The ML algorithm used in CCE is predominantly supervised learning, where the input has been prelabelled. Using CCE as an example, the ML algorithm can produce a precise mapping function to accurately identify polyps by using these prelabelled inputs, colon capsule polyp images, and the paired outputs labelled as “polyp” (Figure 1).

### 2.4. Data Types: Structured and Unstructured Data

Data used and stored in our health care system comprises various formats, for example, graphics, laboratory values, and free-text medical summaries. The type of data is separated into structured data and unstructured data. Structured data is stored and organised in a well-defined manner, often in structured sql databases, spreadsheets, or lists of numbers or categories (e.g., list of names, diagnosis coding, hospital numbers, and laboratory values) that can be analysed by using statistical methods (e.g., addressing a regression or classification question).

The unstructured data type does not have that predefined structure. Without the structured format, they are stored in their raw unstructured form, usually in large text files or nonstructured datasets. They are also categorised as qualitative data, making it more difficult to collect and analyse. This includes images, audio, and video in the form of texts designed to categorise data into different classifications.

Supervised ML algorithms usually require structured data (e.g., videos in which all images are correctly labelled with all relevant classifiers, such as polyps, diverticula, inflammation, residue etc.).

If unstructured data is to be used in machine learning, specialised techniques, such as deep learning, that rely on vast amounts of data would be required. However, that is often unavailable due to confidentiality concerns or the need for more procedures to generate those datasets. Once unstructured data can be used, then applications in predictive analytics could benefit the most [16]. 

Therefore, today, we are looking at using structured data to conduct machine learning and unstructured data to infer from it by using the AI system (e.g., by providing an 80% probability of a mucosal structure being a polyp).

### 2.5. Machine Analysis of the Images

**Figure A1:**
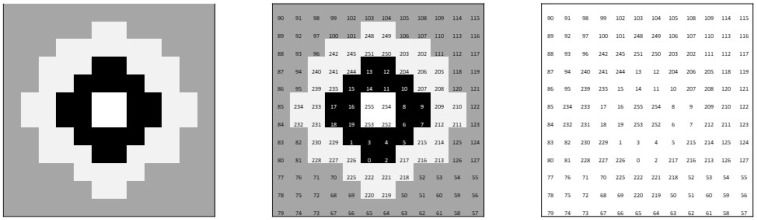
An example of mapping locations in the images to the pixel value as part of machine analysis of the picture.

Images are digitalised into one (black and white image) or many grids of numbers (colour image) (see Appendix A, Figure A1 for a graphical demonstration of the idea). Instead of one grid of numbers representing each pixel, the colour images are represented in three grids (red, green, and blue (RGB)) stacked together. Each pixel’s magnitude represents the corresponding colour brightness in each grid. In practice, a single 224 × 224 pixel colour image would generate 150,528 numbers or features for each image (See Appendix A, Figure A1, a simplified graphical representation of the concept). This demonstrates the immense volume of data incorporated in all the sequences of images within a colon capsule video, which the AI system will have to process to produce the desired output. To overcome this, instead of using the raw data from the images as the input features, the experts adopt a set of handcrafted features manually engineered by the experts. The selected handcrafted features have an enormous impact on the ML and depend on the task to be addressed. For example, simple features like edges, corners, or colour can be used [17].

## 3. The Concept of Neural Network and AI Training

### 3.1. Neural Network 

Due to the large quantity of data, the number of parameters, the spatial information between each pixel and the complexity of the data structure, deep learning (DL) models were created to organise these complex features into architectural layers called neural network (NN) building blocks.

**Figure 2:**
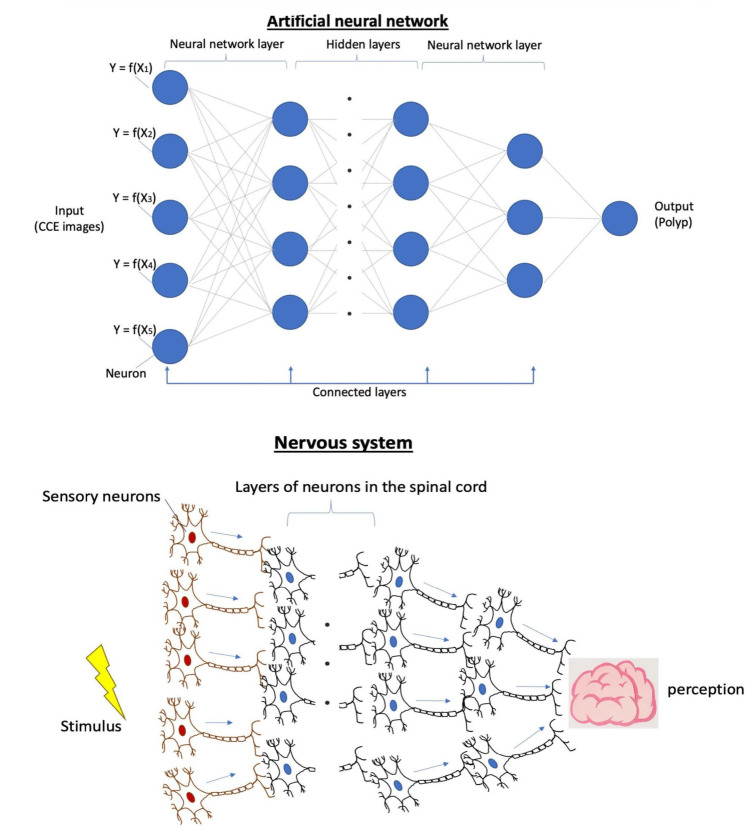
A dense neural network demonstrates the layers’ architecture compared with the nervous system model.

These networks are made up of numerous neurons, each acting as an individual miniature machine learning system (e.g., miniature regression models). A set of neurons, which take the same input, are organised into a layer. These neurons process inputs by using linear combination methods, and the layer’s parameters generate an output which is then fed into the next layer. This process is repeated until the final output is delivered, and it is similar to our nervous system [15]. In addition, there are layers between the first input layer and the last output layer, called hidden layers. The number of hidden layers varies depending on the complexity, function, and associated defined output of the neural network [18] (see Figure 2).

The main difference between NN and traditional ML models is that NN works directly from unstructured, raw data instead of handcrafted features. Whereas traditional machine learning algorithms require an expert to select the problem’s relevant characteristics, NN can automatically perform the feature engineering task.

### 3.2. Convolutional Neural Network

A convolutional neural network (CNN) is mainly designed to process images, and its application is popular in medical fields, such as radiology and endoscopy. It is designed to address two difficulties that a standard neural network encounters when processing images. First, even though a neural network could include and organise many parameters into these dense hierarchical layers, each parameter (neuron) would only be allocated simultaneously to one or a small number of pixel locations. Taking the highly variable positions of the object in a practical image, the number of neurons required is enormous; this inevitably prolongs the processing time considerably. The second issue is that the standard neural network cannot record the spatial information in the image as it flattens the image (the parameters of each pixel organised in specific spatial orders) into a roll of numbers (vector) (see diagram in the Appendix A for more information) [18].

**Figure 3:**
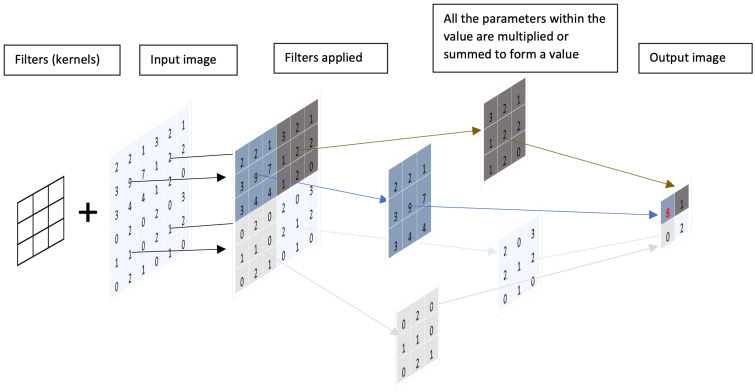
This simplified diagram shows how CNN processes the parameters from an image by using filters (kernels) to condense the parameters to a smaller output to preserve the spatial information and improve the handling speed, as the parameters are analysed in patches rather than individuals.

Consequently, CNN uses the convolutional layers to resolve these issues by using convolutional filters (kernels) (Figure 3). These filters comprise small groups of parameters that are multiplied and summed in patches. The output of each patch is placed relative to the position of the input patch in a new smaller grid. For example, an area of interest (e.g., a polyp on a colon capsule image) could represent a high-value number on the smaller grid.

The output of each convolutional layer can be fed into the next layer as an “image input”. In this sequence, each pixel in the next convolutional layer represents a patch of pixels inputted from the previous layer. After going through various layers of repeated processing, the CNN will be able to see the overall larger patches of the images and ultimately produce output probabilities of the image category [19].

**Figure 4:**
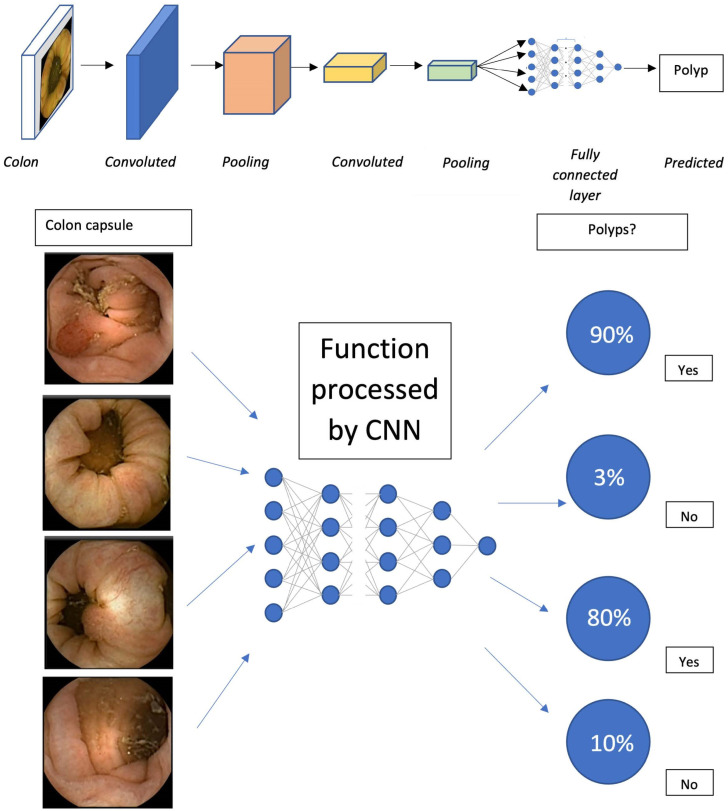
The simplified overall CNN layers in identifying polyps in the CCE video and the flowchart demonstrate the CNN used in the colon capsule video to predict polyps, for example, accurately.

For example, the pixels in the first layer of CNN will form basic features such as small points, lines, and ridges from the raw pixel on the input image. These pixels are then combined again in the successive few layers, by using kernels, into simple shapes such as circles, squares, and large dots. This process repeats until the input data goes deeper into the layers. Suppose a specific combination of shapes or features representing a lesion is present in the deeper layer. In that case, the neurons in that layer will eventually fire the processed features to the final layer that predicts the class of the object (e.g., polyp in the image with a probability score (Figure 4)).

### 3.3. The AI Training, Optimisation and Validation Process

The convoluted neural network models or approximates an accurate mapping function between the inputs and outputs. This requires a slow process of training.

First, the CNN is given a training dataset, a set of data examples for the model to learn and map the function that correlates the inputs to the outputs. In the training set, the difference in error between the CNN’s prediction and the training set’s label will be computed as “loss”. Loss is a numerical value that determines how close the CNN predicted outputs are to the true outputs. After each run of the same training set, the CNN will update its parameters to reduce the loss, called the optimisation step. The CNN will then be evaluated on a validation dataset to assess its performance periodically. It is important to note that the validation dataset was not exposed to the CNN during training and instead only used for validation without modifying the values of the CNN, i.e., it was not being trained.

Hyperparameters are study-specific optional components or parameters in the training programme that trains the model. They are defined manually by the user before the model is trained. They shape the model’s behaviour as part of its performance optimisation by impacting its structure or complexity [20]. They are applied in the training loop in the form of different training configurations to tune the model or algorithm being trained. This is subclassified into two types [14]:

1. model hyperparameters that focus on the architecture of the model (e.g., number of layers in the CNN); and
2. training hyperparameters that determine the training process (e.g., the training rate).

These above steps form a training loop that allows the CNN model to learn generalised and accurate functions from the training sets. At the same time, progress is intermittently validated through the validation datasets. Finally, the model will be examined on a test dataset once the performance is fully optimised and validated. This is an entirely “unseen” set used at the end of the development of the CNN model to confirm its generalised performance on these final sets of data samples.

**Figure 5:**
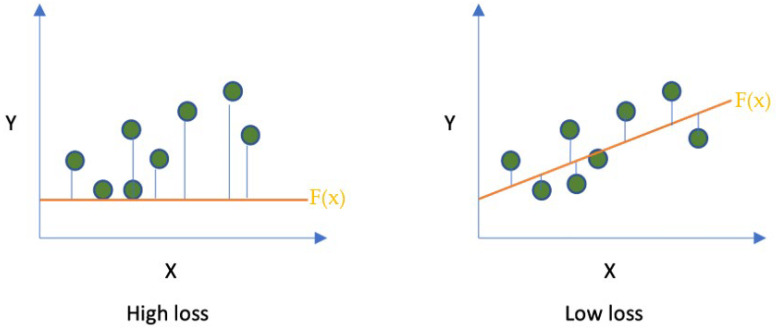
This uses simple linear regression models to demonstrate high and low loss when comparing the predicted output from AI against the true output.

In the training loop, the performance of the CNN is assessed by comparing the predicted output produced by the CNN against the true output. Low-value loss is desirable in machine training. Therefore, the training loop aims to discover a function with the best-fitted parameters to minimise the loss across all the training datasets. This can be illustrated in a simple statistical linear regression example, as shown in Figure 5 [14].

### 3.4. Consequences of Overfitting and Underfitting Data

During machine learning, a balance in the loss needs to be found when conducting a training loop. When the CNN is overtrained (e.g., in an extended training period), it leads to overfitting. This is due to the CNN model memorising irrelevant features, including the background noise from the training set, which is common for these specific patients but not relevant to the finding. Then, the overall accuracy of the validation set starts to deteriorate. The solutions to overcome overfitting include

1. the application of a larger dataset, although in medical imaging, that might not be possible or very costly;
2. modification of the model to a simpler version; and
3. the utilisation of techniques such as regularisation and data augmentation. These methods empower the AI model to learn and preserve the general observations only, allowing the extrapolation of what it has learned to new unseen data.

**Figure 6:**
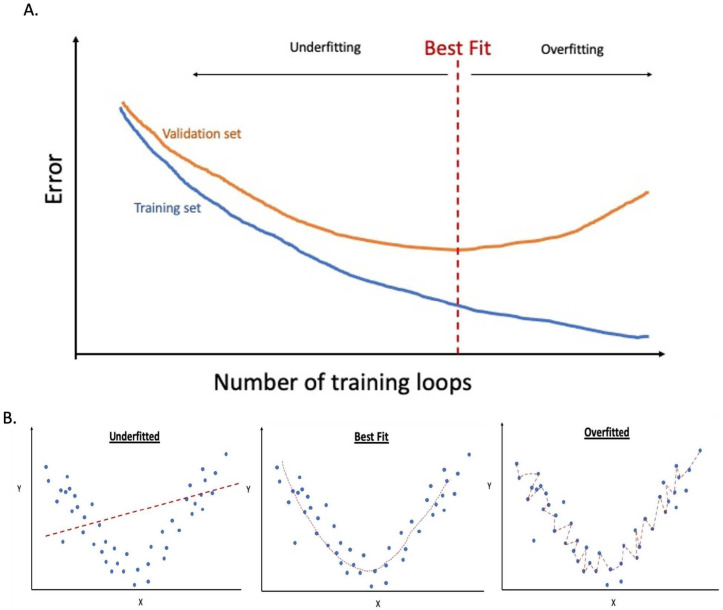
Graphs demonstrate overfitting and underfitting. Graph (**A**) compares the overall error against several loops conducted in the training sets. It shows the error in the validation set uptrends when overfitting occurs while the error in the training set continues to downtrend as the function memorises all the background noise and nonspecific details in the training sets. Finally, graph (**B**) demonstrates the underfitted, best fit, and overfitted concepts by using a simple best-fit trend lines model.

On the other hand, underfitting is equally damaging as this arises from needing to be able to capture the underlying function of the data due to a lack of exposure to the training sets (inadequate training, see Figure 6A) or because of a low complexity of the model (see Figure 6B). Therefore, achieving an appropriate fitting remains one of the significant challenges in this field.

The final step of training an AI is using the a completely new test set to determine the AI model’s overall performance. In a classification problem, measures such are sensitivity, specificity, accuracy, and precision are usually used. Moreover, other global measures such as the receiver operating (ROC) curve or area under ROC curve (AUC) are widely used to compare methods because they do not depend on any threshold.

## 4. Process of Colon Capsule Endoscopy Video Analysis

The American Society of Gastrointestinal Endoscopy (ASGE) and, more recently, its European equivalent (ESGE), have suggested credentialing standards and curriculum for CE reading early on [21]. At the same time, it is known that not only experience in GI endoscopy but concentration capacity and fatigue can also interfere with the outcomes of CE reading [21]. Although there is, to date, no scientific proof or guidelines to indicate the optimal way to read a CCE video, reviewing CCE videos poses extra challenges that are absent in small bowel CE (SBCE) reading. Prolonged segmental delays compound by the to-and-forth, spiraling movement of the capsule in the caecum and proximal colon, the capsule’s bullet-type propulsion in more “muscular” distal colonic segments compound with the colour and turbidity of the luminal fluid, requires time, focused attention, and accurate landmarking for proper evaluation and video review [22].

CCE reading should be performed during protected time slots to maintain high standards and remain a thorough and diligent process, just like any other endoscopic procedure. Admittedly, amassing reading experience can reduce reading times; however, the official time allocated for review/landmarking of a CCE video should be at least 45–65 min for the first/prereaders and at least 25–35 min for the validators on average [21]. The CCE reading time required depends on the cleansing level, colon anatomy, and transit times. Unfortunately, these factors are not predictable. However, it becomes evident that methods to reduce those times and efforts, such as AI, have to be found to reduce the burden on experts and, more broadly, adopt CCE.

Without those methods, the first step should be a quick preview of the entire video: This can be done by using a fast reading (QuickView) mode with both camera heads simultaneously. Next, one should look at the total length of time the capsule needed to go through the colon. Then, they need to identify the landmarks (caecum, hepatic and splenic flexures, and rectum/anus/excretion of capsule).

The second step is a proper review of the images. As the colon capsule is designed to have two cameras, they are represented by yellow and green. Starting from the yellow or green camera but one camera alone, followed by the other camera at a frame rate between 8 and 15 pictures per second. A different approach is advisable if the passage time is short or too long. Often, the capsule tends to stagnate in colonic segments as the colon’s muscular, propulsive mechanism is usually weaker than the SB’s propulsive mechanism. If that occurs, the frame rate could be increased. On the contrary, a short video means that the capsule has gone through the colon quickly, and there are fewer frames to view, so our rate of frames per minute could be decreased by using the scroll wheel (scroll button) on the computer mouse. This often happens in the transverse colon, where the passage time can be quick; a lower (pre)reading speed is advised in this segment to avoid missing lesions.

The last step is reporting the findings. A detailed review of the marked suspected lesions images (thumbnails) that uses white light and virtual chromoendoscopy for characterisation is used when applicable. Each relevant image is annotated and attached by using the hospital reporting or documentation system. The report should finalise all the findings, colonic and extracolonic, transit times, significant findings, diagnosis, and recommendations [22,23].

The optimal frame rate for a thorough colon investigation without any risk of missing lesions remains unanswered. Introducing prucalopride as part of the booster regimen to improve the overall procedure completion rate is being examined. This regimen reduces both the transit and reading times. However, this also potentially increases the risk of missing lesions as the capsule speeds through the colon. More robust data on the frame rate or the minimum length of the video is undoubtedly required in future studies [24,25].

## 5. Evidence-Based Literature Review of AI and CCE

### 5.1. AI in Colon Capsule Endoscopy in the Literature

AI in colon capsule endoscopy is a new field of interest. Recently, Afonso et al. [26] analysed 24 CCE exams (PillCam^®^COLON 2) performed at a single centre between 2010 and 2020. From these video recordings, 3635 frames of the colonic mucosa were extracted, 770 containing colonic ulcers or erosions and 2865 showing normal colonic mucosa. After optimising the neural architecture of the CNN, their model automatically detected colonic ulcers and erosions with a sensitivity of 90.3%, specificity of 98.8%, and an accuracy of 97.0%. The area under the receiver operating characteristic curve (AUROC) was 0.99. The mean processing time for the validation dataset was 11 sec (approximately 66 frames/s).

Saraiva et al. [2] used CCE images to develop a deep learning (DL) tool based on a CNN architecture to detect protruding colonic lesions automatically. A CNN was constructed by using an anonymised database of CCE images collected from 124 patients. This database included images of patients with protruding colonic lesions, normal colonic mucosa, or other pathologic findings. A total of 5715 images (2410 protruding lesions, 3305 normal mucosa or other findings) were extracted for CNN development. The area under the curve (AUC) for detecting protruding lesions was 0.99. The sensitivity, specificity, PPV, and NPV were 90.0%, 99.1%, 98.6%, and 93.2%, respectively. The overall accuracy of the network was 95.3%. This DL algorithm accurately detected protruding lesions in CCE images.

Atsuo Yamada et al. trained a deep CNN system based on a Single Shot MultiBox Detector by using 15,933 CCE images of colorectal neoplasms, such as polyps and cancers [27]. They assessed performance by calculating areas under the receiver operating characteristic curves, along with sensitivities, specificities, and accuracies by using an independent test set of 4784 images, including 1850 images of colorectal neoplasms and 2934 normal colon images. The AUC for detecting colorectal neoplasia by the AI model was 0.90. The sensitivity, specificity, and accuracy were 79.0%, 87.0%, and 83.9%, respectively, at a probability cutoff of 0.35.

Hiroaki Saito et al. [28] used a database of 30,000 VCE images of protruding lesions from 290 patient examinations to develop a CNN model. The CNN model developed from this database was 90% sensitive and 79% specific when identifying test images containing protruding lesions. In addition, subset analyses evaluating model performance for different lesions demonstrated that the model was 86% sensitive for detecting polyps, 92% sensitive for detecting nodules, 95% sensitive for detecting epithelial-based tumours, 77% sensitive for detecting submucosal lesions, and 94% sensitive for identifying protruding venous structures, such as varices. 

Nadimi et al. developed a CNN for the autonomous detection of colorectal polyps; their CNN was an improved version of ZF-Net, a CNN using a combination of transfer learning, preprocessing and data augmentation [29]. They created an image database of 11,300 capsule endoscopy images from a screening population, including colorectal polyps (any size or morphology, N = 4800) and normal mucosa (N = 6500). Their CNN model resulted in an even better performance with an accuracy of 98.0%, a sensitivity of 98.1%, and a specificity of 96.3%. (See Appendix A Table A1 for the summary of the above results)

### 5.2. AI Assessment of CCE Bowel Cleansing

In a pilot study conducted by Buijs et al., a nonlinear index based on the pixel analysis model and a machine learning algorithm based on the support vector machines with four cleanliness classes (unacceptable, poor, fair, and good) were developed to classify the CCE videos of 41 screening participants [30]. The results of both models were compared to cleanliness evaluations by four CCE readers. The ML-based model classified 47% of the videos in agreement with the averaged classification by CCE readers, compared to 32% by the pixel analysis model. In addition, the ML-based model was superior to the pixel analysis in classifying bowel cleansing quality due to a higher sensitivity to unacceptable and poor cleansing quality.

In another study [31], a CAC score, defined as the colour intensities’ red over green (R/G) ratio and red over brown (R/(R + G) ratio, was developed. Bowel cleansing evaluation for each CCE frame was defined as either adequately or inadequately cleansed. Four-hundred-and-eight frames were extracted. Two hundred sixteen still frames were included in the R/G set and 192 in the R/(R + G) set. Regarding the R/G ratio, a threshold value of 1.55 was calculated, with a sensitivity of 86.5% and a specificity of 77.7%.

Regarding the R/(R + G) ratio, a threshold value of 0.58 was calculated with a sensitivity of 95.5% and a specificity of 62.9%. The two proposed CAC scores based on the ratio of colour intensities have high sensitivities for discriminating between “adequately cleansed” and “inadequately cleansed” CCE still frames. Their study showed that CAC scores to assess bowel preparation quality based on a colour intensity ratio of red and green pixels on still images is feasible and rapid (see Appendix A Table A2 for the summary of the above results).

## 6. Challenges of Utilising AI in Endoscopy Video Settings

### 6.1. Understanding the Input Data Used by the AI

One of the main challenges of the deep neural network is the need to understand what signals the model has extracted from the input to draw up the association between the input data and the predicted output. As the AI creates its problem-solving methods, the process is entirely independent of the programmer. An example would be utilising AI to predict fractures on ankle x-rays. The AI can correctly predict fractures based on identifying the arrows that the radiographers drew to indicate the area of interest instead of detecting the discontinuation of the outline of the bone. However, the model drew a conclusion based on nonmedical signals, and the outcome was considered accurate even though it was entirely incorrect. This is a classic example of accidentally fitting confounders rather than the true signal [32,33].

### 6.2. The “Black Box” or Uninterpretable AI Algorithm

**Figure A2:**
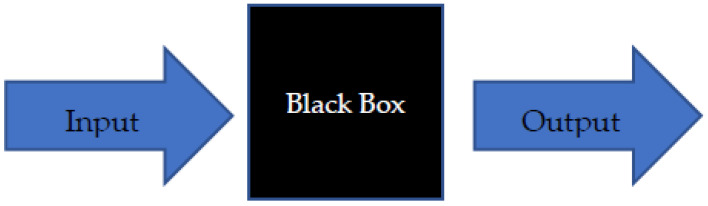
The intrinsic method or behaviour of the AI code in the model is uninterpretable, like a black box with no transparency.

With the complexity of the deep learning neural network, it is very challenging to interpret the AI’s processing methods before arriving at the final output, which is referred to as the neural network black box. The more complex the neural network is, the more accurate but less interpretable it becomes. For example, a student could come up with the answer to a mathematical question without showing any working steps. As a result, it is not easy to understand how the student reaches the solution in the end, which leads to concern about understanding the principles. The need for more clarity and interpretability in these neural networks becomes a significant obstacle in the progression of AI development in the medical field (see Appendix A Figure A2 for a graphical representation of the concept).

Moreover, poor interpretability implies more challenging adjustments to the model for improvement. To overcome this, approaches such as involving a multidisciplinary team to review the false positive and false negative results predicted by the model and testing the model on an external database are adopted [34].

### 6.3. Poor Differentiation between Correlation versus Causation

In addition, the AI model will not be able to differentiate the correlation or causation association between the input and output data. A good example is an AI model correlating the increasing number of drowning cases in the swimming pool with the growing ice cream sales at the entrance in the summer. Therefore, it concludes that growth in ice cream sales causes an increase in drowning incidents in the swimming pool when we know that both of these factors correlate to the hot weather in the summer.

### 6.4. The Importance of Data Quality

In the context of artificial intelligence in CCE, data quality is more important than the neural network algorithm or data engineering techniques. “Garbage in, garbage out” is commonly used in artificial intelligence engineering. This refers to the fact that the chosen data should be high quality, reliable, consistent and reproducible. Unfortunately, in CCE, a wide variation in quantifying the quality of bowel preparation and the bubble effect among experts is a good example. The lack of accurate definitions for these components compromises the data quality and remains problematic for AI development in the field of CE.

### 6.5. Generalisability and “No One Size Fits All”

In addition, sampling strategies and training practices, such as single-institution data, small geographic area sampling, or other approaches, can create unintentional bias and reduce generalisation. For example, a CCE’s AI developed based on an English population’s colon images might not apply to an Asian population. This concept is equivalent to sampling error in statistical terms. Therefore, the feasibility and accuracy of the AI to adapt to various medical imaging techniques in diverse geographical and racial populations still requires further exploration and examination in future studies.

One of the potential solutions is the possibility of sharing datasets among different countries to contribute to building an AI with a large, heterogenous, and multinational super algorithm that allows accurate data processing from any dataset in the world. However, the harmonisation of images is similarly essential. Moreover, in the context of multiinstitutional data sources, there is a potential risk for variable equipment, protocols, etc., which can equally affect the accuracy of the AI outputs [35].

## 7. Future of AI in Gastroenterology

Accurately analysing capsule endoscopy is a time-consuming task for clinicians depending on the comfort and expertise of the reader [35,36]. Using AI can reduce that time by helping clinicians during analysis and reduce diagnostic errors due to human limitations such as biases and fatigue. This would ultimately lead to more time for clinicians to focus on training and diagnosing pathologies. This wireless and patient-friendly technique, combined with rapid reading platforms and the help of artificial intelligence, will become an attractive and viable choice to alter how patients are investigated in the future within gastroenterology [37]. With the growth of telemedicine stepped up by the COVID-19 pandemic, a large part of specialist care will continue to be performed remotely. As CCE becomes more established, it has enormous potential in telemedicine settings.

With that in mind, there are concerns about future jobs in the gastroenterology sector being replaced by AI automation. However, instead of job replacement, we anticipate the shift toward job displacement by focusing more resources on the tasks that are not easily automated, such as clinician and patient interaction, more complex procedures, complex decision-making, education, and training. In addition, new jobs or industries, such as medical machine learning engineering, might be required in the future medical health system.

The human–AI partnership would suggest that the machine cannot be used alone. Furthermore, overdependence on AI would undoubtedly lead to deskilling, especially in the form of cognitive work, such as polyp detection and recognition. Therefore, the key to integrating AI into gastroenterology should focus on balancing AI automation and the personal care we value for our patients to provide an efficient and cost-effective endoscopy service in the future [38,39,40].

## 8. Conclusions

In the future, AI is expected to offer multiple beneficial applications in GI disease risk stratification, lesion recognition and assessment, diagnosis, and treatment. The progress in the last decade suggests that AI-aided CCE will be available soon and radically transform medical practice and patient care. Understanding the fundamentals and the basic concepts of machine learning technology will not only strengthen the trust in AI among clinical professionals but prevent any unintended pitfalls in AI applications in future clinical practice. This may allow future AI refinement or optimisation with a multidisciplinary team approach.

With the current ethical uncertainty and challenges, future multicentre, randomised trials, which validate AI models, should focus on answering the fundamental question of whether AI models can enhance physician performance safely and reliably. In the end, a robust multidisciplinary collaboration among physicians, computer scientists, and entrepreneurs is required to promote AI’s clinical use in medical practice [38,39,40].

## Figures and Tables

**Table 1:** Relevant technical terminology in machine learning.

Terminology:	Definitions:
Artificial intelligence (AI)	It is a technology that enables a machine to simulate a human’s natural intelligence and behaviour.
Machine learning (ML)	It is a subfield of AI that focuses on how a computer system develops its intelligence to predict the result of unseen data accurately.
Example	It is a single pair of input-output data used in training an ML algorithm. It includes paired features and labels in each example. A set of examples form a dataset.
Features	This is the input data that is fed into the machine learning system. For example, in CCE, the visual properties of the images (input data) are processed in the form of a collection of numbers to form features for the ML system.
Labels	The precise output data used to compare with the prediction (the predicted output generated by the ML system). In CCE, labels are the annotations of polyp by an expert reader. Then, the AI-predicted results are verified against these labels.
Prediction	The output data produced from an unseen input by the ML system that has learned from many training samples.
Training loop	This is a repeated training process to allow sufficient machine training to take place. This is performed on numerous sets of input–output data (examples) in a training set.
Training dataset	This comprises a set of examples that are used for the ML system to learn the function that connects the features to the labels.
Validation dataset	This comprises a set of examples that are only used periodically to assess and tune the hyperparameter values that were trained on the training set.
Test dataset	This contains a set of examples that the ML system has never been exposed to. It tests the ML system’s generalisation performance to unseen data.
Deep learning	A type of machine learning model that is formed by numerous layers of neural network and allows the features to be organised into hierarchical layers. The major difference from traditional machine learning is that the features and relations are directly learnt from the input data (end-to-end learning) to produce the prediction.
Hyperparameters	These are the parameters used to control the learning procedure and train the model. This is predetermined before the training set. The selection of hyperparameters includes the size of the sample set and the number of layers in the neural network. The hyperparameter tuning process involves changing the training configurations and this takes place when the model is evaluated on a validation dataset.
Convolutional neural network (CNN)	It is a type of neural network that is designed for visual imagery. It uses convolutional filters (kernels) to build a shared-weight architecture that includes layers of fully connected neural networks.
Classification	A form of supervised learning and the goal of the model is to match that input with predefined categories at the output. For example, the CCE ML algorithm classified the lesions into polyp or cancer, which are predefined categories.
Overfitting	A phenomenon occurs when the model starts to learn all the detailed features, such as the background noise and “memorise” the training set (tightly fitted to the training set). This happens when the error in the validation dataset starts to deteriorate due to poor generalisation to new data (the model only works well in the training examples as it has memorised all the details).
Underfitting	A phenomenon occurs when the model cannot obtain a good fit of the trend to the dataset due to a lack of training or the model’s design is too simple to fit a complex trend.
Regularisation	Techniques used to address overfitting by commanding the model to learn and retain some generalisation during the training procedure.

## Data Availability

Not applicable.

## Appendix A

**Table A1:** Summary of CNN performance for detection of colonic lesions.

Study	No. of Images	Colonic Lesion	Normal Colonic Mucosa	Sensitivity	Specificity	Accuracy of the Network	AUROC for Detection of Protruding Lesion
Afonso [26]	3635	770	2865	90.3%	98.8%	97.0%	0.99
Saraiva [2]	5715	2410	3305	90.0%	99.1%	95.3%	0.99
Atsuo Yamada [27]	4784	1850	2934	79.0%	87.0%		0.902
Hiroaki Saito [28]	17,507	7507	10,000	90.0%	79.0%		0.911
Nadimi, E.S [14]	1695	4800	6500	98.1%	96.3%	98.0%	

**Table A2:** Summary of the two studies on AI assessment of CCE bowel cleanliness.

Study	Type of AI	Number of Videos/Frames Analysed	Level of Agreement AI with Readers, %	Sensitivity	specificity
Buijs [30]	Non-linear index model SVM mode	41 videos 41 videos	32% 47%	_ _	_ _
Becq [31]	R/G ratio R/(R+G) ratio	216 frames 192 frames	- -	86.5% 95.5%	77.7% 62.9%
